# The Editor's Letter-Box

**Published:** 1913-02-01

**Authors:** Margaret Breay

**Affiliations:** Hon. Secretary (Cert. St. Bartholomew's Hospital). The Society for the State Registration of Trained Nurses, 431 Oxford Street, London, W.


To Thomas Hayes, Esq.,
Clerk to the Governors
of St. Bartholomew's Hospital.

Sir,—I am directed to say that it has been brought to the notice of this Society that a resolution has recently been passed by the committee of St. Bartholomew's Hospital depriving the nursing staff of freedom of speech concerning their registration by the State, which affects their educational, economic, and social status, thus denying them the power, within the institution, of discussing their own professional affairs. I shall be obliged if you will confirm or deny this statement.—I am, Sir, yours faithfully,

(Signed) Margaret Breay,
Hon. Secretary
(Cert. St. Bartholomew's Hospital).

The Society for the State Registration of Trained Nurses,
431 Oxford Street, London, W.,
January 20.

St. Bartholomew's Hospital,

